# Rotavirus vaccination in the US: a systematic review of vaccination coverage and completion

**DOI:** 10.1080/21645515.2020.1794440

**Published:** 2020-08-26

**Authors:** Parinaz K. Ghaswalla, John D’Angelo, Remon Abu-Elyazeed

**Affiliations:** GSK, Philadelphia, PA, USA

**Keywords:** Rotavirus vaccines, completion, compliance, coverage, United States

## Abstract

A systematic literature review of Medline and Embase databases was conducted to describe rotavirus (RV) vaccine coverage for a complete series, timing of receipt of all doses in the series, and predictors of RV vaccination coverage in the US for two licensed RV vaccines (RV1, RV5). Nine publications were included in the review. RV vaccination coverage rates of under 80% suggest RV vaccines are underutilized relative to the Healthy People 2020 target and other childhood vaccines. About 50–90% of children initiating RV vaccination complete the series and coverage for a complete series is lower for black and Hispanic children (vs. whites), uninsured or Medicaid insured (vs. privately insured), and for foreign-born (vs. US-born) children. Series completion is significantly greater in children receiving DTaP, RV1 (vs. RV5), and for those receiving routine care from a pediatrician. There is a need to design and implement better RV immunization strategies for US children.

## Introduction

Rotavirus (RV) is the most common cause of severe pediatric gastroenteritis that generally infects children by the age of 5.^1^ Since 2006, RV vaccination has been recommended in the United States (US) by the Advisory Committee on Immunization Practices (ACIP) to protect children against RV gastroenteritis.^2^ Prior to the introduction of RV vaccines in the US, there were annually about 410,000 physician visits, 205,000–272,000 emergency department (ED) visits, and 55,000–70,000 hospitalizations due to RV infections.^2^ RV vaccination has been very impactful in preventing severe RV gastroenteritis in children, with the number of RV-positive tests performed across the US decreasing 74–90% compared with the pre-vaccine baseline.^1^ Indirect benefits of vaccination, such as reduction in RV-related hospitalizations among unvaccinated populations, have also been observed.^3^ Thus, RV vaccination has contributed to important public health benefits based on the reduced clinical and economic burden of RV disease in the US.^4^

Several elements can influence receipt of vaccination, such as access to health care administration considerations (dosage form/presentation, co-administration with other vaccines, number of doses), payment (covered by insurance or not), acceptability (parent/caregiver attitude), and recommendations (local, state, federal).^5^ The Centers for Disease Control and Prevention (CDC) monitors vaccination coverage, defined as the estimated percentage of people who have received specific vaccines, to understand how well communities are protected from vaccine-preventable diseases.^6^ In 2016, RV vaccination coverage for the complete series of RV vaccine in the US was 74.1%,^7^ which is below the 80% target coverage for RV vaccines in the Healthy People 2020 objectives.^8^ It is worth noting that national vaccine coverage rates do not provide insight about the timing of receipt of doses measured as compliance rates, i.e., whether the recommended number of doses are received as per the recommended dosing schedules, or whether completion rates may be different for the two currently licensed RV vaccines in the US (RotaTeq, pentavalent RV vaccine [RV5], Merck & Co., Inc., USA, and Rotarix, monovalent RV vaccine [RV1], GSK, Belgium), one requiring two doses (RV1) and the other requiring three doses (RV5) as per the respective US prescribing information (PI) (Table 1). While the two RV vaccines have different dosing schedules in the PI, the ACIP recommends a harmonized dosing schedule for RV5 and RV1 (Table 1^2^) and is the recommended dosing schedule in CDC’s childhood immunization schedule.^11^ Furthermore, compared to the coverage of other childhood vaccines in the US, such as Diphtheria-Tetanus-acellular Pertussis (DTaP), coverage for RV vaccine lags behind despite having similar dosing schedules.^12^ For instance, between 2006 and 2016, annual DTaP vaccine coverage rates among children aged 19–35 months ranged from 93.7% to 96.2%, whereas RV vaccine coverage levels increased but remained lower than DTaP vaccine coverage (43.9% in 2009 to 74.1% in 2016).^7,13,14^ The age restrictions and lack of a catchup recommendation contribute to lower RV vaccine coverage compared to DTaP coverage in the US.^12^ To help improve vaccination coverage for RV vaccines, it is important to understand vaccination series completion and compliance rates, for the two currently licensed RV vaccines in the US (RV1 and RV5). To address barriers to higher RV vaccine coverage rates and develop effective interventions to improve suboptimal coverage rates, it is also necessary to review the factors that influence coverage, completion, and compliance rates. One publication summarized and reviewed RV vaccine coverage, adherence to age recommendations and related RV-vaccine experience data for the US, but was restricted to the first three years of post-licensure data.^15^

**Table 1.** Dosing schedules of RV vaccines licensed and recommended in the US

	RV1 (two-dose series)		RV5 (three-dose series)	
	PI schedule^9^	ACIP	PI schedule^10^	ACIP
Recommended ages for doses	-	At 2, 4 months	-	At 2, 4 and 6 months
Minimum age for first dose	6 weeks	6 weeks	6–12 weeks	6 weeks
Maximum age for first dose	-	14 weeks and 6 days	-	14 weeks and 6 days
Interval between doses	≥ 4 weeks before maximum age	≥ 4 weeks	4–10-week intervals before maximum age	≥ 4 weeks
Maximum age for last dose	24 weeks	8 months and 0 days	32 weeks	8 months and 0 days

ACIP: Advisory Committee on Immunization Practices; PI, prescribing information; RV, rotavirus; RV1: Rotarix, GSK, Belgium; RV5: RotaTeq, Merck & Co., Inc., USA

This systematic literature review was conducted with the goal to collate and describe for a population of US children (i) RV vaccine coverage rates, (ii) RV vaccine series completion rates for all recommended doses in the series, (iii) RV vaccine series compliance to the recommended dosing schedules (ACIP-harmonized schedule and US PI), and (iv) socio-demographic, health-care utilization, access-related and other factors associated with RV vaccine series completion and compliance.

## Methods

This review was conducted according to guidelines in the Cochrane Handbook for Systematic Reviews of Interventions.^16^ In-line with these guidelines, we developed a search strategy and study eligibility criteria prior to conducting the review. Following this, searches were performed and retrieved publications were assessed for eligibility in a two-phase screening process by two reviewers. Data were extracted from the final list of eligible publications. As the final step, we synthesized key findings from the data. The review methodology is detailed below.

### Search source and strategy

We searched PubMed and Embase in May 2018. Search terms “(rotavirus OR rotavirus vaccines) AND (compliance or completion or adherence or predictors)” were used. Filters for the English language and human studies were applied. We further restricted the searches to studies conducted in the US, because the implementation of vaccination programs can vary by country, and published from 2006 onwards, i.e., from the year the first RV vaccine (RV5) was licensed for use in the US.

### Study selection criteria

Article eligibility criteria were established *a priori*. The “STrengthening the Reporting of Observational Studies in Epidemiology” (STROBE) statement was used to define the review eligibility criteria.^17^ Inclusion criteria were: (1) studies that included children identified from population-level databases, including national surveys or administrative claims; (2) studies conducted in the US; (3) vaccine intervention that included either RV vaccine licensed in the US (RV5 or RV1); (4) measured outcomes that included RV vaccination coverage rates or series completion, defined as receipt of all recommended RV vaccine doses, or series compliance to the recommended dosing schedule for RV vaccines, based on both the ACIP-harmonized schedules and the respective US PI as shown in Table 1, and/or factors associated with series completion or compliance; and (5) any observational study design that assessed completion or compliance rates relative to RV vaccine coverage. Review articles, efficacy or immunogenicity trials, effectiveness, and modeling studies were excluded.

### Screening and selection

After the searches were performed, the identified publications were screened in two phases by two reviewers (PKG, JDA). The first phase included screening of titles and abstracts of all publications based on the eligibility criteria and was followed by the second phase which consisted of reviewing the full-text publications. Any discrepancies in article inclusion were resolved through a discussion between authors.

### Data collection and reporting

From the selected studies, one reviewer extracted data on study population, data source, study setting, vaccine type, rates of and factors associated with RV vaccine series completion and compliance, then a second reviewer checked the quality of the extracted data. Data were extracted into Microsoft Excel 2016. In this article, we present an overview of the key findings.

## Results

### Overview of included studies

**Figure 1.**
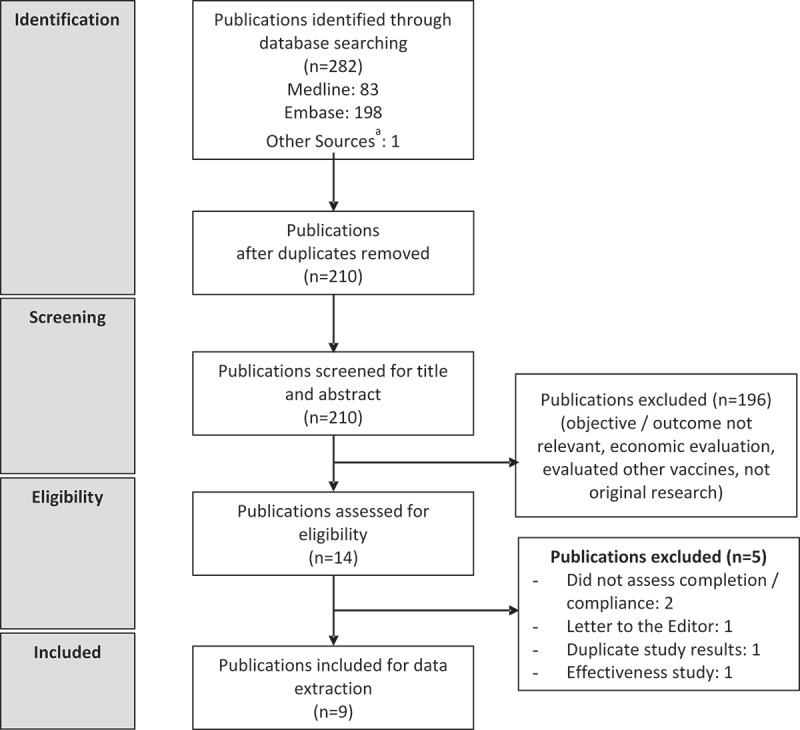
Systematic review flow diagram

^a^Hill et al^7^

The database search identified 282 publications of which 210 were screened based on their title and abstracts after removing the duplicates. Of these 210, 14 full-text publications were assessed for eligibility after excluding studies with irrelevant outcomes such as cost analyses, DTaP studies, efficacy or safety studies, and other exclusions as detailed in Figure 1, according to the Preferred Reporting Items for Systematic Literature Reviews and Meta-Analyses (PRISMA) checklist guidelines.^18^ A total of nine articles were included in the review. Table 2 summarizes the methodological characteristics and the main findings from the included studies.^7,19-26^ Six were retrospective cohort studies from large administrative claims databases^19-22,24,25^ and three were based on national surveys conducted annually by the CDC.^7,23,26^

**Table 2.** Summary of included studies assessing RV vaccine series completion/compliance with schedules in the US

Author; Publication Year	Data Source; Study Period	Sample Size	RV Vaccine Completion Rate	RV Vaccine Compliance Rate	Factors impacting series completion/compliance
Calnan M. et al^19^	Medicaid administrative claims data from four states; May 2008 – April 2013	Cohort 1: 658,219 met PI criteria Cohort 2: 675,963 met ACIP criteria	Cohort 1: 55% (RV1) vs. 44% (RV5), *p* < .0001 Cohort 2: 60% (RV1) vs. 47% (RV5), *p* < .0001	Cohort 1: 55% (RV1) vs. 25% (RV5), *p* < .0001 Cohort 2: 57% (RV1) vs. 46% (RV5), *p* < .0001	*Increased likelihood*: use of RV1 (vs. RV5); DTaP vaccination completion, Fee-for-service insurance (vs. other/unknown) *Decreased likelihood*: attending non-pediatric specialties (vs. pediatric specialty)
Eisenberg D. et al^20^	Administrative claims data from 14 commercial health plans (HealthCore); August 2008 – January 2011	Cohort 1: 162,614 met PI criteria Cohort 2: 164,596 met ACIP criteria	Cohort 1: 85% (RV1) vs. 76% (RV5), *p* < .05 Cohort 2: 85% (RV1) vs. 78% (RV5), *p* < .05	Cohort 1: 69% (RV1) vs. 54% (RV5), *p* < .05 Cohort 2: 81% (RV1) vs. 66% (RV5), *p* < .05	-
Hill A. et al^7^	Survey data from the National Immunization Survey-Child; 2016	14,988	74%^a^	-	*Decreased likelihood*: Black and Hispanic (vs. White), living below federal poverty level, Medicaid or uninsured (vs. private insurance)
Krishnarajah G. et al^21^	Administrative claims data of managed care enrollees (OptumInsight); January 2009 – March 2010	55,584	91% (RV1) vs. 83% (RV5), *p* < .001	75% (RV1) vs. 60% (RV5) compliant to PI; *p* < .001 83% (RV1) vs. 76% (RV5) compliant to ACIP criteria	*Increased likelihood*: use of RV1 (vs. RV5), pediatrician as provider type (vs. family physician), month of series initiation March-June (vs. January), geographic region Midwest, South, West (vs. Northeast)
Krishnarajah G. et al^22^	Medicaid administrative claims data from 10–13 states (MarketScan); May 2008 – June 2012	Cohort 1: 673,956 met PI criteria Cohort 2: 695,612 met ACIP criteria	Cohort 1: 65% (RV1) vs. 46% (RV5), *p* < .0001 Cohort 2: 74% (RV1) vs. 49% (RV5), *p* < .0001	Cohort 1: 65% (RV1) vs. 31% (RV5), *p* < .0001 Cohort 2: 69% (RV1) vs. 46% (RV5), *p* < .0001	*Increased likelihood*: Receipt of DTaP vaccine, use of RV1 *Decreased likelihood*: Black or Hispanic race (vs. White)
Kurosky S. et al^23^	Survey data from the National Immunization Survey; 2012	11,710	90% (RV1)^b^; 81% (RV5)^b^	77% (RV1); 70% (RV5)	-
Lanes S. et al^24^	Administrative claims data from commercial health plans (HealthCore); February 2006 – November 2012	272,142	78% (RV5, enrolled at birth through 1^st^ birthday); 77% (RV5, enrolled by 6 weeks of age and no minimum follow-up)	-	-
Panozzo C. et al^25^	Administrative claims data, commercial insurance (MarketScan); January 2006 – September 2010	594,117	87% (RV1) vs. 79% (RV5), *p* < .001	83% (RV1); 78% (RV5)	*Increased likelihood*: Receipt of DTaP vaccine, pediatrician provider type (vs. family physician) *Decreased likelihood*: mother’s age <25 years, having siblings <10 years old (vs. no siblings), residing in rural and small urban areas
Varan A. et al^26^	Survey data from the National Immunization Survey; 2010–2012	52,441	65%^a^	-	*Increased likelihood*: non-Hispanic Asian or non-Hispanic other, child’s age ≥24 months, maternal education ≥12 years, income-to-poverty ration ≥400%, region of residence in Midwest or South *Decreased likelihood*: Foreign-born (vs. US-born), lacking health insurance, not being first-born, having 2 healthcare providers

ACIP: Advisory Committee Immunization Practices; DTaP: Diphtheria-Tetanus-acellular Pertussis; PI: prescribing information, RV1: Rotarix, GSK, Belgium; RV5: RotaTeq, Merck & Co., Inc., USA

^a^Includes RV1 or RV5

^b^Completion rate at 24 months of age

### Vaccine coverage, completion and compliance rates from national surveys

A complete RV vaccine series (defined as either ≥2 doses of RV1 or ≥3 doses of RV5) was reported for 74.1% of children aged 19–35 months based on provider-reported vaccination data from the National Immunization Survey (NIS) for 2016 – a small decline from the previous year (73.2% in 2015).^7^ Data from the same type of NIS survey, but for 2010–2012, showed that about 65% of children completed an RV vaccine series.^26^ The results from these two studies show that while the coverage of the RV vaccine series has increased over time, this rate is still well below the Healthy People 2020 target of 80%. In a third study, RV completion and compliance rates for 2010–2012 were determined separately for RV1 and RV5.^23^ About 90% of children completed the two-dose series of RV1 compared to 81% who completed the three-dose series of RV5 by 24 months of age. Also, 77% and 70% of children were compliant to the RV1 and RV5 series, respectively, by receiving all required doses on time according to the ACIP-harmonized schedule.^23^

### Vaccine completion and compliance rates for commercially-insured children

RV vaccination completion and compliance rates based on large claims databases for commercially-insured children have been determined in four studies with periods ranging from 2006 to 2012.^20,21,24,25^ Three of these studies compared RV vaccine completion and compliance rates for RV1 and RV5 and found that completion rates, measured as the percentage of patients receiving all required doses, were significantly higher for patients who received RV1 compared to RV5 (87% vs. 79%; 91% vs. 83%; 85% vs. 78%; *p* < .05).^20,21,25^ Another study estimated the completion rate of RV5 at 78% among children with continuous enrollment from birth through the first year of life but did not provide a comparison to RV1.^24^ Two of these studies also determined compliance rates as per the respective US PI and the harmonized ACIP dosing schedules, which addresses mixed series completion using both RV1 and RV5.^20,21^ In both studies, compliance rates per PI schedule were higher for RV1 compared to RV5 (69% vs. 54%, *p* < .05;^20^ 75% vs. 60%, *p* < .001^21^). Compliance rates as per ACIP schedule in the two studies were also higher for RV1 compared to RV5 (81% vs. 66%, *p* < .05;^20^ 83% vs. 76%, *p* < .001^21^).

### Vaccine completion and compliance rates for Medicaid children

Two studies estimated RV vaccination completion and compliance rates among large cohorts of children covered by Medicaid programs.^19,22^ Medicaid is a joint federal and state program in the US, that helps with medical costs for individuals with low incomes and limited resources.^27^ In both studies, completion and compliance were assessed based on the respective US PI and the harmonized ACIP dosing schedules. In the PI cohort, more children who received RV1 completed the series compared to those who received RV5 (55% vs. 44%, *p* < .0001;^19^ 65% vs. 46%, *p* < .0001^22^) and were compliant with the PI schedule (55% vs. 25%, *p* < .0001;^19^ 65% vs. 31%, *p* < .0001^22^).

### Factors associated with vaccine completion and compliance

In addition to dosing schedules and vaccine characteristics, several factors influenced vaccine completion and compliance including social and geographic characteristics, family characteristics, access and provider characteristics, and immunization characteristics. These factors had a statistically significant impact on vaccine completion and compliance and are examined further.

#### Social and geographical characteristics

Coverage for a complete RV series was lower among black children and Hispanics than among white children (67.2% and 73.0% vs. 77.3%, *p* < .05), and among children living below the federal poverty level compared to children living at or above the poverty level (65.5% and 78.2%, *p* < .05).^7^ A relatively small but significant difference in RV vaccine series completion by geographic region showed higher completion rates in the Midwest, South, and West regions of the US compared to the Northeast.^21^ This association between region of residence and RV vaccine series completion was not found to be significant in another study.^25^ However, the latter study showed that children residing outside of metropolitan areas were less likely to complete the RV vaccine series.^25^ In another study, data from 2010 to 2012 from the NIS showed a huge disparity in RV series completion rates between 19–35-month-old foreign-born and US-born children (15.7% vs. 65.7%, *p* < .001).^26^

#### Family characteristics

Mother’s age and number of siblings were found to be associated with RV series completion so that children born to younger mothers (<25 years; 73.9%) and children with one or more siblings less than 10 years of age (72.4–79.5%) were less likely to complete the series than mothers aged 30–34 years (81.5%) and children with no siblings (82.2%), respectively.^25^

#### Access/provider characteristics

Provider type was found to be a significant factor associated with RV series completion in two studies.^21,25^ In one study, children who received routine care from a pediatrician were more likely to complete their series compared to children who were receiving routine care from a family physician (risk ratio [RR] = 1.13; 95% confidence interval [CI] = 1.11–1.14).^25^ Similarly, the second study reported that children were less likely to complete their series when the immunization provider type was family practice compared to pediatricians (RR = 0.88; 95%CI = 0.87–0.89).^21^ RV vaccine coverage for the completed series also varied by health insurance status with lower levels of coverage for uninsured children or those covered by Medicaid compared to those with private insurance (59.9% vs 68.7% vs 80.7%, *p* < .05).^7^

#### Immunization characteristics

*Type of RV vaccine*. Children receiving the two-dose RV1 were found to be more likely to complete the vaccine series compared to children receiving the three-dose RV5 across three studies.^19,21,22^ Two of these studies used data from a population of Medicaid-insured beneficiaries^19,22^ and one study assessed RV vaccine completion rates in a managed care population.^21^

*Receipt of DTaP vaccine*. An overlap exists between the recommended schedules for DTaP and RV vaccination, and three studies demonstrate a significant association between DTaP receipt and RV vaccine series completion.^19,22,25^ One of these studies tested the strength of the DTaP association with series completion by running two multivariable analyses – for a 2006 birth cohort and a 2009 birth cohort. While significant in both years, the strength of this association declined from 2006 (RR = 1.47; 95%CI = 1.32–1.63) to 2009 (RR = 1.24; 95%CI = 1.19–1.29).^25^ Medicaid children vaccinated with DTaP had a higher likelihood of compliance with RV vaccination (RR = 17.8; 95%CI = 17.4–18.3).^22^ In a population of Medicaid children, those who completed the DTaP vaccine series were 11.82 times more likely to be compliant with an RV vaccine series (RV1 and RV5) compared to those who did not complete DTaP vaccination.^19^

*Other*. The association between month of series initiation and RV vaccine series completion was only evaluated in one study. This study evaluated the first 6 months of the year and children who initiated an RV vaccine series in the months of March, April, May, and June were shown to have significantly higher RV vaccine series completion rates compared to those initiating the series in January.^21^

## Discussion

This comprehensive review of available literature on RV vaccination coverage, completion and compliance rates, and factors influencing RV vaccination, suggests that RV vaccination remains underutilized in US children. Although only nine studies were eligible for inclusion, the consistent finding across these studies is that a sizable proportion of US children either do not get vaccinated against RV or do not receive vaccinations according to the recommended dosing schedules.

Despite the increase in coverage for RV vaccine over the years to 74.1% in 2016, it is still underutilized in US children relative to the Healthy People 2020 target coverage of 80%.^7,8^ RV vaccine coverage rates are lower than observed rates for most recommended childhood vaccines (≥90%) in 2016.^6,7^ Data also showed that about 50-90% of the US children initiating RV vaccination complete the RV series and compliance rates with the recommended schedules are even lower.^19–22,24,25^ In addition, compliance and completion per PI and ACIP recommendations were found to be significantly greater among children receiving RV1 than those receiving RV5 among studies that are nationally representative and included both commercial and Medicaid insured children.^19,21,22^ While it may seem obvious that completion and compliance with a two-dose series are easier to achieve than with a three-dose series, other factors are contributory, making vaccine coverage a complex issue.

RV coverage for a complete series is lower for black and Hispanic children (vs. whites),^7^ uninsured or Medicaid insured (vs. privately insured),^7^ and for foreign-born (vs. US-born) children.^26^ Series completion and compliance are significantly greater in children receiving DTaP vaccination,^19,22,25^ RV1 compared to RV5 vaccination,^19,21,22^ and for those receiving routine care from a pediatrician compared to family physicians.^21,25^ The finding of higher RV vaccine completion rates for those receiving DTaP can be explained by the overlapping recommended dosing schedules for RV and DTaP vaccines, as children presenting to a provider’s office to receive DTaP vaccination at 2, 4 or 6 months of age, also have the opportunity to receive RV vaccines.^28^ Because the receipt of DTaP vaccination was associated with higher rates of completion and compliance for the RV vaccination series,^19,22,25^consideration should be given to administering the first dose of RV vaccine as soon as possible after 6 weeks of age, along with DTaP vaccination as feasible, to ensure induction of protection prior to exposure to natural RV infection.^29^ Additional factors that influence RV vaccination coverage include region in the US,^21^ poverty level,^7^ family characteristics such as mother’s age, and number of siblings.^25^These disparities indicate that improvements are needed in access to and delivery of age-appropriate immunization.

The search of online databases for this review was performed in May 2018. Since then one additional article has been published on this topic by Aliabadi et al.^12^ Their analysis of RV coverage, timing of initiation, and completion of the vaccine series among children enrolled in seven US medical institutions that serve as active gastroenteritis surveillance sites were consistent with the findings of the studies included in this review in that coverage for RV vaccines was found to be lower than DTaP vaccine and factors that were associated with higher likelihood of RV vaccine completion were recent birth years (2013–2016) and higher maternal education. Preterm birth, African-American race, and public or no insurance were associated with lower odds of RV vaccine completion and regional differences were also observed.

This review focused on compliance, completion, and vaccine coverage rates, which are all considered important determinants of successful vaccine implementation, particularly with multi-dose vaccines.^30^ A reduction in any of these parameters means that an individual or population has not received the full protection that is intended to be delivered through vaccination. This has adverse consequences for the individual, the at-risk population, and society at large such as reduced community immunity, decreased quality of care, and reduction in work productivity.^31^ While the effectiveness of partial vaccination with RV5 in preventing RV-related hospitalizations and ED visits has been demonstrated,^32^ it should also be noted that these estimates reflect short-term protection as most children in the study went on to complete the full three-dose series. In addition, there were inconsistencies reported in the effectiveness against RV-related outpatient visits such that one dose of RV5 was found to be more effective than two doses (100% vs. 40%).^32^ Another study also demonstrated the benefits of incomplete vaccination in terms of reduced RV-disease burden compared to an unvaccinated cohort.^4^ However, there are no clinical trial data for the efficacy of partial RV vaccination and the ACIP does not recommend incomplete vaccination. CDC’s reporting of national RV vaccination coverage is also based on full series completion (i.e., ≥2 doses of RV1 or ≥3 doses of RV5).^7^

Even though RV vaccine programs differ in settings outside of the US, evidence from these settings tends to support high and sustained RV1 vaccination coverage. In England, a significant reduction in direct healthcare costs was reported in the first year of vaccine introduction and as high RV1 vaccination coverage (93% and 88% for one and two doses, respectively) was achieved.^33^ Similarly, in Norway, an 86% decrease in RV gastroenteritis hospital cases was observed in children <5 years in 2016 compared to 2014–2015.^34^ A high national coverage rate for RV1 vaccine series was documented in the first year after introduction (coverage rates of 89% and 82% for one and two doses, respectively). Among fully RV-vaccinated children, 98% received both doses within the upper age limit of 16 weeks and 90% received both doses according to the recommended schedule in Norway.^35^ In Canada, RV1 vaccine series initiation ranged from 83.2% to 91.3%, with full series completion increasing each year of the program (Aug 2011-Jul 2014) from 73.0% to 78.5% and 84.2%.^36^ In a study conducted in Austria, incomplete RV vaccination (RV1 or RV5) emerged as a risk factor for vaccine failure (OR 5.7; 95%CI: 4.2–7.8) underscoring the significance of complete vaccination.^34^

Our review has several limitations. The generalizability is limited to the US and not applicable to other countries. While it describes reports on compliance, completion, and coverage to define a good level of implementation and impact of RV vaccine, there is limited evidence to support the link between these factors and clinical outcomes such as the reduction of RV gastroenteritis or RV-related hospitalizations, and that was not within the scope of this review. The link is difficult to assess for technical reasons, as cohorts with different vaccination statuses (completely vaccinated, incompletely vaccinated, unvaccinated) should be followed over time. Most of the studies included in this review have overlapping time periods and identified factors using one combined study period. It is likely that the factors may change over time, but none of the studies specifically addressed this question.

## Conclusion

Despite ACIP recommendations to vaccinate children against RV, vaccine coverage in the US is not optimal. Further efforts are necessary to identify those children who are not reached through current vaccination strategies and to assess interventions to improve completion of RV vaccine series. In addition to factors such as the environment, combination with other vaccines, and timing of vaccination, data from multiple studies in different populations indicate that an RV vaccine with fewer doses may help improve vaccination coverage and, presumably, disease protection through higher rates of completion and compliance.

## Abbreviations

ACIP: Advisory Committee on Immunization Practices
CDC: Centers for Disease Control and Prevention
CI: confidence interval
DTaP: diphtheria-tetanus-acellular pertussis
ED: emergency department
NIS: National Immunization Survey
PI: prescribing information
RR: risk ratio
RV: rotavirus
RV1: Rotarix, GSK, Belgium
RV5: RotaTeq, Merck & Co., Inc., USA

## Trademark statement

Rotarix is a trademark owned by or licensed to the GSK group of companies. RotaTeq is a trademark of Merck & Co., Inc.

## Appendix

**Plain Language Summary**

*What is the context?*

- Currently, two vaccines against rotavirus can be administered to infants in the US: Rotarix (RV1), a two-dose-series vaccine, and RotaTeq (RV5), a three-dose-series vaccine.
- Disease control is promoted by obtaining high vaccination coverage and receipt of the entire vaccine series within the recommended vaccination schedule.

*What is new?*

- This systematic literature review summarizes published rates of rotavirus vaccine coverage, series completion, and compliance to the recommended dosing schedule among US children, and factors associated with rotavirus vaccine series completion and compliance in this population.
- It shows that immunization against rotavirus is suboptimal with reported vaccination coverage below the Healthy People 2020 target of 80%.
- Factors associated with rotavirus vaccine series completion include the child’s socioeconomic characteristics, insurance status and type, vaccine provider type, receipt of diphtheria-tetanus-pertussis vaccination, and rotavirus vaccine type.

*What is the impact?*

- A better awareness of the gaps in rotavirus vaccination coverage and series completion, and understanding of factors associated with vaccine series completion or compliance to dosing schedule, could help improve protection against rotavirus disease and its complications among children in the US through the improvement of rotavirus immunization strategies.
